# Hospital dental practice in special patients

**DOI:** 10.4317/medoral.19553

**Published:** 2013-10-13

**Authors:** Javier Silvestre-Rangil, Francisco J. Silvestre, Fernando Espín-Gálvez

**Affiliations:** 1 Profesor asociado del Departamento de Estomatología. Universitat de València; 2 Profesor Titular del Departamento de Estomatología. Universitat de València. Jefe de la Unidad de Estomatología del Hospital Universitario Dr. Peset, Valencia; 3 Jefe del servicio de Cirugía Maxilofacial del Complejo Hospitalario Torrecardenas, Almeria

## Abstract

Dental patients with special needs are people with different systemic diseases, multiple disorders or severe physical and/or mental disabilities. A Medline search was made, yielding a total of 29 articles that served as the basis for this study, which offers a brief description of the dental intervention protocols in medically compromised patients. Dental treatment in patients with special needs, whether presenting medical problems or disabilities, is sometimes complex. For this reason the hospital should be regarded as the ideal setting for the care of these individuals. Before starting any dental intervention, a correct patient evaluation is needed, based on a correct anamnesis, medical records and interconsultation reports, and with due assessment of the medical risks involved. The hospital setting offers the advantage of access to electronic medical records and to data referred to any complementary tests that may have been made, and we moreover have the possibility of performing treatments under general anesthesia. In this context, ambulatory major surgery is the best approach when considering general anesthesia in patients of this kind.

** Key words:**Hospital dentistry, special patients, medically compromised patients.

## Introduction

The dental management of patients with special needs, whether medically compromised or with severe disabilities, is sometimes complex and requires a multidisciplinary and integral approach. The hospital is therefore the ideal setting for treating these patients, due to the availability of specialized technical and human resources found in such centers.

The hospital setting allows dental practice to interrelate with other clinical and surgical specialties, and offers the support of central or core services that can facilitate diagnosis and treatment. Such interrelation is particularly relevant with the Departments of Oral and Maxillofacial Surgery and Stomatology; indeed, the Hospital Dental Surgery Unit sometimes forms part of such Departments (1).

Although most forms of dental care in special patients are provided by the public primary care services, there are certain situations in which hospital resources are needed in patients of this kind. Examples include the use of general anesthesia in the treatment of certain patients with serious physical or mental disabilities when behavioral control proves very difficult, when dental treatment must be completed in a single session, or when there is a strong possibility of medical emergencies developing during the intervention. In this sense, the Hospital Dental Surgery Unit can serve as a link or bridge between the primary care setting and the specialized Department of Oral and Maxillofacial Surgery (1).

Hospital Dental Surgery Units should carry out integral activities such as the diagnosis and treatment of oral mucosal lesions, the diagnosis and treatment of orofacial pain and of temporomandibular joint disease, oral surgery, the dental treatment of medically compromised patients such as those subjected to radiotherapy for head and neck tumors, the use of diagnostic and management protocols in patients programmed for organ transplantation, and the integral dental care of patients with severe disabilities using general anesthesia. Likewise, these Units should facilitate dental interconsultations and attend dental emergencies in long-stay hospitalized patients (1,2).

Logically, these Dental Surgery Units should be structured in accordance to the reference or recruitment population of the centers to which they belong, and in concordance with the available resources, the objectives of the Health Department, and the range of services to be offered to users.

## Material and Methods

An electronic Medline search was made, based on the following key words: special patient, special care and hospital dentistry. The search included review articles and adequately designed clinical trials. We excluded clinical cases, clinical trials with methodological shortcomings, and articles unrelated to special patients. A total of 28 articles were identified, as well as one book chapter, which were used as the basis for the present study.

## Medical risk evaluation in hospital dental practice

dental patients with special needs are people with different systemic diseases, multiple disorders or severe physical and/or mental disabilities. In these patients we need thorough knowledge of the buccodental impact of their background disease, and must provide dental care that does not adversely affect their general health. In order to provide specific and integral management in these cases, appropriate means and personnel are needed (3).

Before starting any dental treatment, we need a correct anamnesis, medical records and interconsultation reports in order to know as much as possible about the problems of the patient. In this context, the hospital setting offers the advantage of access to electronic medical records and to data referred to any complementary tests that may have been made (3).

The medical risk also must be evaluated before starting any treatment in these patients. To this effect we use the ASA scoring system developed by the American Society of Anesthesiologists (4). This classification contemplates 6 scores according to the patient background illness.

Specifically, ASA I includes healthy patients able to walk up at least one flight of stairs without problems, and who suffer little or no anxiety. Very young or very old patients are excluded.

ASA II in turn corresponds to patients with mild systemic disease, including smokers without chronic obstructive pulmonary disease (COPD); slight obesity; slightly elevated blood pressure controlled with medication; thyroid gland disorders; type II diabetes controlled with diet or drugs; asthmatics who occasionally use inhaled medication; stable chest pain (except if under extreme stress); very anxious patients with a history of fainting episodes in the dental office; patients with myocardial infarction in the previous 6 months but without symptoms; and patients over 65 years of age.

ASA III refers to patients with serious systemic disease limiting daily life activities, such as individuals with type I diabetes; morbid obesity; chest pain with clinical manifestations in response to minor physical exertion; systolic blood pressure between 160-194 mmHg and diastolic blood pressure between 95-99 mmHg; patients subjected to chemotherapy; COPD (bronchitis and emphysema); swelling of the ankles (heart failure); hemophilia; frequent asthma attacks or seizures; and patients with myocardial infarction in the previous 6 months but who still present symptoms.

ASA IV corresponds to patients with serious systemic disease that poses a constant threat to life. This group includes uncontrolled diabetes; patients with chest pain or shortness of breath while sitting in the absence of physical exertion; individuals unable to walk up a flight of stairs; patients who wake up at night with chest pain or shortness of breath; chest pain that worsens even with medication; patients visiting the dental office with oxygen therapy; individuals with myocardial infarction or stroke in the last 6 months; and individuals with a blood pressure of over 200/100 mmHg. In the case of ASA IV patients, dental treatment should be provided in the hospital setting in order to avoid complications (4,5).

Stress can increase morbidity in medically compromised patients, producing physiological changes (6). It therefore must be regarded as a risk factor in dental treatment, in the same way as very old age, excessive medication use, or the administration of immunosuppressors or anticoagulants. In patients at risk we must ensure good pain control and the use of premedication and sedation techniques to control anxiety (3,6).

## Considerations regarding the dental treatment of special patients

1. Cardiovascular Diseases

Patients with cardiovascular diseases represent one of the most common groups of medically compromised patients seen in dental practice. This group includes patients with hypertension, ischemic heart disease and arrhythmias.

1.1. Arterial Hypertension

Arterial hypertension is defined as systolic blood pressure elevation to 140 mmHg or more, or diastolic blood pressure elevation to 90 mmHg or more, on occasion of at least three different blood pressure recordings. Hypertensive patients can develop complications such as ischemic heart disease, stroke, renal failure, heart failure, blindness, and even malignant hypertension - a serious emergency condition (7,8).

Hypertensive individuals with good blood pressure control can be treated without risk. In these cases we use anxiolytic premedication and make sure that a good local anesthetic technique is used. It is also necessary to monitor patient blood pressure before and after treatment, and to avoid sudden shifting of the dental chair, in order to avoid orthostatic hypotension. In the event of a hypertensive emergency, we can administer 40 mg of furosemide and then add 25 mg of captopril after a few minutes if necessary. Alternatively, nitrites in physiological saline can be administered if we are working in the hospital setting (7,8).

1.2. Ischemic Heart Disease

Ischemic heart disease occurs as the result of partial or complete obstruction of coronary blood flow, often caused by thrombus formation over atheroma plaques, which obstructs the vascular lumen. If obstruction is complete and causes tissue necrosis, myocardial infarction results. In contrast, in the event of only partial coronary occlusion without myocardial necrosis, the patient experiences chest pain (9,10).

In patients who have suffered a cardiovascular event in the previous 6 months, it is advisable to provide only emergency treatment, making use of minimally invasive procedures with a view to eliminating pain. The stress of visiting the dental office should be minimized by keeping the visits short, and offering effective pain management, with a good local anesthetic technique - limiting the administered volume to no more than two anesthetic cartridges (9,10).

1.3. Patients with Antiplatelet and/or Anticoagulation Therapy

In the case of patients receiving antiplatelet medication and who need dental treatment, it could be dangerous to suspend the medication. In any case, the decision to discontinue antiplatelet treatment should be made in coordination with the physician in charge of patient (10). In patients receiving anticoagulant treatment, the international normalized ratio (INR) must be determined – ruling out certain dental interventions when the recorded values is under 3. Local hemostatic measures must be adopted in patients receiving either type of therapy, including the use of hemostatic agents such as oxidized and regenerated cellulose or collagen sponges. In addition, suturing is indicated, applying compression with gauze impregnated in tranexamic acid or epsilon-aminocaproic acid. These drugs can also be prescribed via the oral route, or as oral rinses (9).

1.4. Cardiac Arrhythmias

Cardiac arrhythmias are defined as alterations in heart beat secondary to rhythm, frequency or myocardial contraction disorders. Patients with such problems may be at risk in situations that cause anxiety. Consequently, we should program short visits and simple treatments, and also administer anxiolytic premedication before dental intervention (7,8). In these individuals, vasoconstrictor use in anesthesia should be minimized, and due monitoring of the patient should be ensured at all times. In patients carrying pacemakers, automated defibrillators or other neurostimulatory devices, ultrasound used to eliminate supragingival tartar, and the electrical scalpel, might generate interferences with such devices (10).

In the event of an emergency due to severe arrhythmia, the dental procedure should be suspended, the patient vital signs should be recorded, and oxygen and sublingual nitrites should be administered. In addition, the patient should be placed in the Trendelenburg position, vagal nerve maneuvering may be used, and we should be prepared to perform life support measures and to emergency evacuate the patient if necessary (7,8).

1.5. Prevention of Bacterial Endocarditis

Preventive measures against bacterial endocarditis should be considered in patients at high risk of developing this kind of infection, such as individuals with heart disease and valve prostheses or prosthetic material used to repair heart valves; a history of infectious endocarditis; non-repaired congenital cyanotic heart disease (shunts and ducts); congenital heart defects fully repaired with prosthetic material or devices in the past 6 months; repaired congenital heart disease with residual defects at or near the location of a patch prosthesis or prosthetic device; and heart transplant recipients that have developed valve disease.  Table 1 shows the measures indicated for preventing infectious endocarditis in these patients (7,11).

**Table 1:**
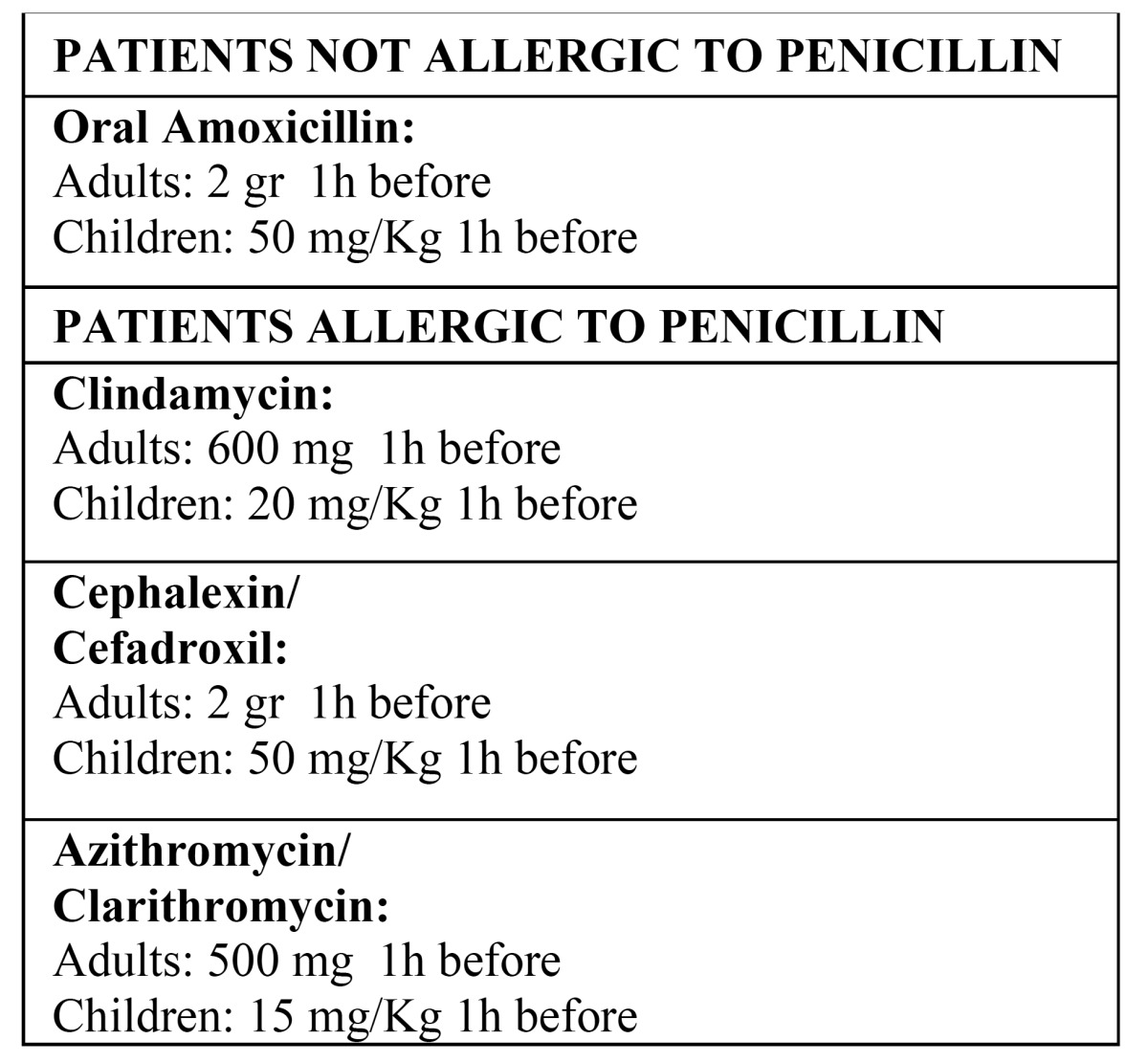
Protocol for the prophylaxis of bacterial endocarditis.

2. Respiratory Disease

Patients with respiratory disease are also commonly seen in hospital dental practice. These individuals include cases of chronic obstructive pulmonary disease (COPD) and asthma.

2.1. Copd

Chronic obstructive pulmonary disease is characterized by chronic and irreversible obstruction of the lower airway. In this context, chronic bronchitis develops when obstruction is caused by excessive mucus secretion se-condary to a chronic bronchial inflammatory process. Emphysema in turn is characterized by distal distension of the terminal bronchioles, with destruction of the alveolar walls. Dental treatment of these patients requires prior evaluation of their current condition and degree of dyspnea. Dental treatment moreover should be avoided during exacerbations of the disease. We must avoid all actions that might further worsen respiratory function; in this context, it would be advisable not to make these patients wait in the waiting room, and we should perform simple treatments, place the dental chair as vertical as possible, and avoid certain techniques such as the use of rubber dams. On the other hand, care is required with sedation and the abuse of antibiotics. The administration of drugs producing dry mouth can further worsen the problem of airway dryness. Lastly, general anesthesia should be avoided as far as possible, and if the patient condition worsens, dental treatment should be suspended (12,13).

2.2. Asthma

Bronchial asthma is an inflammatory disorder characterized by reversible airway obstruction secondary to an increase in bronchiole muscle tone with edema and congestion of the respiratory mucosa, and an increased capacity to respond to certain stimuli (hyperresponsiveness). In these patients we must establish the type of asthma involved and the corresponding triggering factors, with the purpose of avoiding them. Likewise, the patient should be instructed to bring his or her inhaler medication and corticosteroids (if any) to the dental office, since supplementary dosing may prove necessary. Stressful environments are to be avoided, and anxiolytic premedication can be provided. The patient should use the bronchodilator before starting the dental treatment, and the latter must be suspended in the event of any sign of asthma attack. In such a situation, patient positioning should be improved, and airway permeability must be ensured. The patient should be instructed to administer three salbutamol puffs, applying nasal oxygen, and if the condition fails to improve, 100 mg of hydrocortisone and 0.3-0.5 mg of subcutaneous adrenalin can be provided, followed by emergency evacuation (12,13).

3. Renal Function

Renal function is impaired by disease processes that affect the glomeruli, tubules and interstitial tissues of the kidneys. In extreme cases renal function can deteriorate to life-threatening levels. These disorders, referred to as renal failure, may be either acute or chronic, and can ultimately lead to end-stage renal disease (ESRD) (12).

3.1. Renal Failure

In patients with renal failure subjected to conservative management, dental treatment can be provided if a series of recommendations are taken into account, such as restriction of nephrotoxic drug use (in the case of nonsteroidal antiinflammatory drugs (NSAIDs), tetracyclines, aminoglycosides, acyclovir or antihistamines among others), or prolongation of their dosing interval (as in the case of amoxicillin, metronidazole and paracetamol) (12,14). We also should request a complete blood count and coagulation tests, adopting local hemostatic measures if necessary (12). In patients with more advanced-stage renal disease subjected to hemodialysis, antibiotic prophylaxis must be provided in order to avoid infection of the arteriovenous fistula, and dental treatment moreover should be carried out on the day after hemodialysis (12,15,16).

3.2. Renal Transplantation

In kidney transplant patients, and in addition to the above recommendations, it would be advisable to evaluate the patient before transplantation, in order to avoid septic foci and ensure a good buccodental condition. Only emergency treatments should be considered, attempting to avoid infection in the early post-transplantation period. Once the graft has been shown to be stable and the nephrologist agrees, we can offer treatment for these patients, taking into account their tendency towards infection and bleeding. Oral hygiene should be controlled in view of the risk of gingival overgrowth in patients receiving immunosuppressive therapy in the form of cyclosporines. On the other hand, if corticosteroids are administered on a chronic basis, supplementary dosing may be needed. Treatment should also be provided for any oral ulcerations that may develop, and close monitoring is required in view of the important risk of tumor development in these subjects (12,15).

4. Liver Failure

Liver failure or insufficiency is defined as the inability of the liver to perform its synthetic and metabolic functions in the normal physiological context. Liver failure is classified as either acute or chronic - the typical causes being alcoholism and viral infections. Only emergency dental treatment should be provided in patients with active viral hepatitis. As in the rest of special patients, interconsultation with the supervising physician is required, with a detailed clinical history, and careful avoidance of possible cross-infections. Coagulation testing is also indicated, with the adoption of hemostatic measures when planning dental surgery. In turn, the use of hepatotoxic drugs such as paracetamol, tetracyclines, ketoconazole, opiates, monoamine oxidase inhibitors (MAOIs) and barbiturates, among others, should be restricted (17,18).

5. Endocrine Disease

Individuals with endocrine disease represent another group of patients requiring special considerations in relation to dental care. Such diseases include thyroid and adrenal gland disorders, and diabetes.

5.1. Hyperthyroidism

Hyperthyroidism, also known as thyrotoxicosis, is characterized by an increase in thyroid gland hormone secretion, resulting in high blood thyroid hormone concentrations (19,20). In patients with controlled hyperthyroidism, we can provide dental treatment as usual, though attempting to avoid situations of stress and the development of infection. Likewise, in cases of uncontrolled hyperthyroidism, adrenalin use should be restricted, with the avoidance of infections, since emergency situations in the form of thyrotoxic crises could result (19).

5.2. Hypothyroidism

Hypothyroidism is characterized by deficient thyroid hormone synthesis. Controlled patients can receive dental treatment, taking care to avoid acute infections that may lead to clinical decompensation. In decompensated patients we can expect delayed healing, and caution is required when using drugs such as anesthetics, analgesics, barbiturates, hypnotic agents and tranquilizers. It is important to treat emergencies conservatively, and to wait for the patient to be clinically controlled, due to the risk of myxedematous coma. In children with cretinism, the existing mental retardation requires careful behavioral control (19,20).

5.3. Addison’s Disease

In patients with adrenal gland disorders such as Addison’s disease, situations that can cause emotional tension are to be avoided, since their tolerance of stress is lowered. Anxiolytic premedication is thus indicated, with a good local anesthetic technique and interconsultation with the supervising physician, in order to adjust the corticosteroid dosage if necessary. Infectious foci are to be avoided, and the tendency of these patients to develop melanic staining of the oral cavity should be taken into account in relation to possible differential diagnosis (20,21).

5.4. Cushing Syndrome

Prolonged corticosteroid use can produce Cushing syndrome. Our effort in the clinical management of these patients should focus on avoiding the complications of hypertension, hyperglycemia, heart failure, delayed healing and depression. It is important to be careful during surgery, due to the risk of fracture secondary to glucocorticosteroid-induced osteoporosis (20). Likewise, possible corticosteroid supplementing should be evaluated, in view of the poor tolerance of stress in these individuals (21).

5.5. Diabetes Mellitus

Diabetic patients constitute the most important group of individuals with endocrine disorders, and present alterations in carbohydrate, lipid and protein metabolism. Diabetes is attributable to a decrease in the availability or activity of insulin. In patients of this kind it is important to have a good clinical history, since they suffer a range of systemic alterations and moreover receive drug treatments that can complicate the provision of dental care. Individuals with well controlled diabetes can receive normal dental treatment. In such circumstances it is advisable for the patient to follow a normal diet in order to avoid possible hypoglycemic episodes while in the dental office. Such episodes are characterized by anxiety, confusion, drowsiness, restlessness, seizures, paleness, cold and humid skin and tachycardia, and can lead to diabetic coma. Hypoglycemic crises should be treated administering fast-absorbing carbohydrates via the oral route if the patient is conscious, or providing 10% glucose solution via the intravenous route. On the other hand, dental treatment should be carried out in the morning, which is when the endogenous corticosteroid levels are highest and the patient is better able to tolerate situations of stress. Blood glucose measurements are advised before the dental intervention, with the administration of broad spectrum antibiotics to cover the risk of infection, since these individuals are more susceptible to immune system dysfunction and healing difficulties (20,22).

6. Oncological Patients

6.1. Patients Subjected to Radiotherapy

The oncological patients which we can see in the hospital setting include individuals programmed for radiotherapy of the head and neck. These patients require evaluation before the start of radiotherapy, in order to adopt adequate measures referred to prevention and hygiene, prepare protective splints, eliminate septic foci, and perform any necessary dental extractions at least 15 days in advance. After radiotherapy, our work is to treat the short-term complications such as mucositis, possible overinfections of the mucosal ulcers associated with mucositis, and dysgeusia. To this effect, the patients should follow a soft diet without irritants. In turn, 2% lidocaine rinses can be prescribed, and removable dentures should be avoided (23,24). Patients should be instructed to brush their teeth with high fluor concentration toothpaste, and chlorhexidine varnish should be applied every three months to avoid the development of caries secondary to hyposialia. Tooth extractions should be postponed in order to avoid the development of osteoradionecrosis (23).

6.2. Patients Subjected to Chemotherapy

In patients receiving chemotherapy, we should adopt measures referred to prevention and hygiene, and eliminate septic foci from the oral cavity before starting the treatment. During chemotherapy, dental operations should be limited to emergency conditions, using noninvasive techniques, with treatment of the complications such as mucositis and xerostomia. Antibiotic prophylaxis is also advised, since these patients are very susceptible to infection. Furthermore, coagulation tests should be requested before each surgical procedure (23,24). Dental treatment in such individuals should be provided after the chemotherapy cycles have been completed, once the hematological parameters have norma-lized (23,24).

7. Neurological Patients

As regards the neurological patients we may see in dental practice, mention must be made of those with epilepsy, Parkinson’s disease or advanced stage Alzheimer’s disease.

7.1. Epilepsy

In relation to epilepsy, we should avoid treating patients with frequent and uncontrolled seizures until the condition has been brought under medical control. When dental treatment is started, we must avoid triggering factors, particularly stress and anxiety, and anxiolytic agents can be prescribed if necessary. In the event of an epileptic crisis, the patient should be placed lying face up and with the head turned to one side; any material or elements are to be removed from the oral cavity, and mandibular clamping is to be avoided in order to prevent tongue biting (12,25).

7.2. Alzheimer’s Disease

In patients with Alzheimer’s disease we can observe dry mouth secondary to the medication they receive, and deficient oral hygiene. Preventive measures are therefore also indicated, and the relatives should be instructed on how to assist the patient in this regard. We should avoid sudden shifting of the dental chair, in order to avoid orthostatic hypotension. In advanced stages of the neurodegenerative disease, behavioral control of the patient may prove complicated; treatment under general anesthesia therefore may be considered (12,26).

7.3. Parkinson’s Disease

Patients with Parkinson’s disease can present trembling of the facial muscles, making it difficult for them to retain removable dentures, as well as drooling. Once the degenerative disease has started to produce symptoms, early rehabilitation of the oral cavity should be carried out, with the adoption of preventive measures referred to hygiene and bacterial plaque control (12,27,28).

## Hospital treatment under general anesthesia

one of the advantages of hospital dental practice is the possibility of providing treatment under general anesthesia. Although it should only be used as a last resort, in certain special patients general anesthesia represents the only way to provide effective and safe dental treatment (5).

In this context, ambulatory major surgery is the best approach when considering general anesthesia in patients of this kind. It consists of the provision of dental treatment in a session under safe and controlled general anesthesia, without the posterior use of a hospital bed. At the end of anesthesia, the patient is monitored in a waking room, and following recovery is moved to a ward until he or she is able to return home, where postoperative control is subsequently continued through te-lephone contact.

Ambulatory major surgery offers a number of advantages, including lesser aggressivity for the patient, the avoidance of nosocomial infections and of a lack of hospital wards adapted to disabled patients, and a reduction of the economical costs and waiting time. In turn, patient satisfaction is high with this type of surgery, and there is no associated increase in morbidity (5).

The situations in which general anesthesia is indicated include medically compromised patients with problems that make their treatment potentially dangerous if carried out in a conventional dental office; patients unable to collaborate because of physical or mental disabilities, or very young age; patients requiring extensive dental treatment that must be provided in a single session; patients with important craniofacial anomalies and extensive treatment needs; individuals with severe orofacial injuries; and patients who must travel long distances for dental care, where the completion of treatment in a single session is desirable (5,29).
